# Patient-Reported Outcome Measures in Endometriosis

**DOI:** 10.3390/jcm10215106

**Published:** 2021-10-30

**Authors:** Alba Nicolas-Boluda, Anne Oppenheimer, Jerome Bouaziz, Arnaud Fauconnier

**Affiliations:** 1 OneClinic, 75008 Paris, France; anb@one.fr (A.N.-B.); dr.jeromebouaziz@pointgyn.com (J.B.); 2 PointGyn, 78370 Plaisir, France; 3 EA 7285 Research Unit “Risk and Safety in Clinical Medicine for Women and Perinatal Health”, Versailles-Saint-Quentin University (UVSQ), 78180 Montigny-le-Bretonneux, France; anneoppenheimer@yahoo.fr; 4 Department of Reproductive Medicine and Fertility Preservation, Hopital Universitaire Antoine Béclère, 92140 Clamart, France; 5 Centre Hospitalier Intercommunal de Poissy-Saint-Germain-en-Laye, Department of Gynecology and Obstetrics, 78300 Poissy, France

**Keywords:** endometriosis, patient reported outcome measures, quality of life

## Abstract

Patient Reported Outcome Measures (PROM) evoke measurements that allow capturing patients’ perspectives on their condition. In endometriosis care, physicians’ understanding of the effect of the disease and the treatment on patients is often poor. The use of PROMs in endometriosis clinical practice can facilitate patient-provider communication and the implementation of patient-centered care, improve patients’ quality of life, as well as provide a tool for patients’ self-management of the disease. Today, PROMs are extensively used in research and clinical trials, however they are barely used in clinical practice. The development of digital tools facilitating capturing PROMs can contribute to their use by physicians in routine endometriosis care. However, all PROMs are not adapted to be used in routine care in the context of endometriosis. The objective of this study was to present a catalogue of available PROMs for routine endometriosis care and evaluate them according to selected criteria. To do so, we explored the different PROMs currently in the literature. Consequently, 48 PROM were identified as tools used to evaluate various dimensions of the impact of endometriosis on patients. The selected PROMs were evaluated for their potential to be used as a standard in clinical practice in endometriosis. The selected catalogue of PROMs is the starting point for the integration of digital tools to capture PROMs and the development of patient-centered dashboards to be used by patients and clinicians in endometriosis care and self-management to improve care processes, patient satisfaction, quality of life, and outcomes.

## 1. Introduction

Endometriosis is a benign disease characterized by the growth of endometrial-like cells outside the uterine cavity, which induce chronic inflammation. The prevalence of endometriosis is between 5% and 10% on average in women of childbearing age [1,2]. Patients with endometriosis manifest varied symptoms including pelvic symptoms, severe dysmenorrhea, chronic pelvic pain, deep dyspareunia, gastro-intestinal and low urinary tract symptoms, and infertility [3]. Endometriosis symptoms, especially pain, affect patients’ ability to properly function physically and mentally, leading to severe consequences in social interactions and psychological symptoms such as depression. Overall, endometriosis severely affects patients’ quality of life [4,5].

Patient-reported outcome measures (PROMs) are standardized, validated questionnaires that capture patients’ self-perceived symptoms and health status, which includes symptoms, physical and social function, mental health, and overall wellbeing [6]. PROMs aim to measure all aspects related to a patient’s health status, as reported by the patient, without a physician’s influence and interpretation. PROMs have been used extensively as research tools in clinical trials, and their results have been confirmed as reliable and consistent across different types of populations. Recently, PROMs have been used as a support for care and in developing new care models [7]. The use of PROMs in health care is a key to the transformation towards patient-centered care, an essential value of health systems [8]. PROMs can evaluate the impact of clinical interventions from patients’ perspectives, which is an essential aspect because of the often poor understanding by clinicians of the effect of the patient’s disease and prescribed treatment on their life [9]. The impact of PROMs in managing endometriosis is particularly reinforced in endometriosis because the patients’ perceptions of the disease can differ significantly from that of the physicians [10]. Indeed, two patients with the same clinical profile do not have the same perception of their disease, and the impact on their quality of life is not necessarily the same [10].

The routine use of PROMs in managing endometriosis could significantly improve care. The use of aggregated PROM data from individual patients can be used to audit and evaluate the impact, appropriateness, quality, and performance of healthcare services [11]. PROM tools can also be used in routine clinical practice at the individual patient level during their care process, allowing clinicians to complement their classical approach of medical consultation and physical examination with the patients’ view of their disease, providing patient-centered care, an approach that facilitates patient-clinician communication [12]. This approach is relevant to endometriosis because PROMs can identify issues that are not easily raised in consultation such as the impact on the sexual quality of life. PROMs also provide an opportunity for clinicians to discuss their patients’ expectations of a given treatment and identify incompatibilities or unrealistic expectations. PROMs are therefore useful as shared decision-making tools [13]. PROMs can also be employed for screening specific conditions (e.g., depression and anxiety) and alerting clinicians to a problem not previously mentioned by the patient [14]. In endometriosis, PROMs could therefore be used to optimize the patients’ care process. Knowing what matters to patients can help optimize the design and delivery of health care [6]. Outside the clinical research context, there is currently no standardized method for patients to communicate their needs, symptoms, treatment responses, and undesired side-effects. The US Food and Drug Administration (FDA) has therefore recommended PROMs for evaluating the repercussions of endometriosis and evaluating its therapeutic strategy [15]. 

PROMs have been used in endometriosis for some time but only as research tools, with studies using different PROM tools to describe each aspect of the disease. One of the main roadblocks to the implementation of PROMs in clinical practice is the feasibility of implementation. Clinicians are often reluctant to the use of PROMs in routine care due to the fear of additional burden and workload. This is true if considering collecting PROMs manually using pen and paper. The recent development of clinical tools facilitate the collection of PROMs and their routine use in clinics. PROMs can be collected digitally prior to the consultation at the patient’s home or in the waiting room and results can readily be displayed to the clinician on a dashboard ready to be used as a support during consultation. However, all PROM tools are not adapted to be used in routine clinical practice nor be used in digital tools. Different features in PROMs need to be evaluated prior to their selection for routine clinical practice in endometriosis:

1. Parsimony—extremely lengthy PROM tools are not compatible with routine care. In endometriosis care, different outcomes related to the disease need to be measured. The use of lengthy PROMs to measure each of the outcomes results in a significant time investment for the patient.
2. Digitalization—the digitalization of PROMs is an essential leverage for their integration in eHealth. The capacity of capturing data in real time, allowing immediate transfer of information from the patient to the clinician, allows their use during consultations.
3. Unidimensionality—PROMs can be used as digital biomarkers. To do so, only PROMs that provide an unidimensional score will be able to be used in algorithms or as an aid to medical decision.
4. Content validity—Often, PROMs are developed in the general population and used in specific populations without prior validation. Before their use in clinical practice, PROMs need to validated in the target population (in this case, patients diagnosed with endometriosis).
5. Relevance—selected PROMs should measure outcomes that matter to patients.

There is therefore little consensus on which PROM tools are the best for endometriosis and in particular for their use in routine clinical care. To determine the most appropriate PROM tools for use in endometriosis care, we explored the types of PROMs currently employed in the literature, classified them according to the type of outcomes measured, and evaluated the above-mentioned features. 

## 2. Materials and Methods

**Figure 1:**
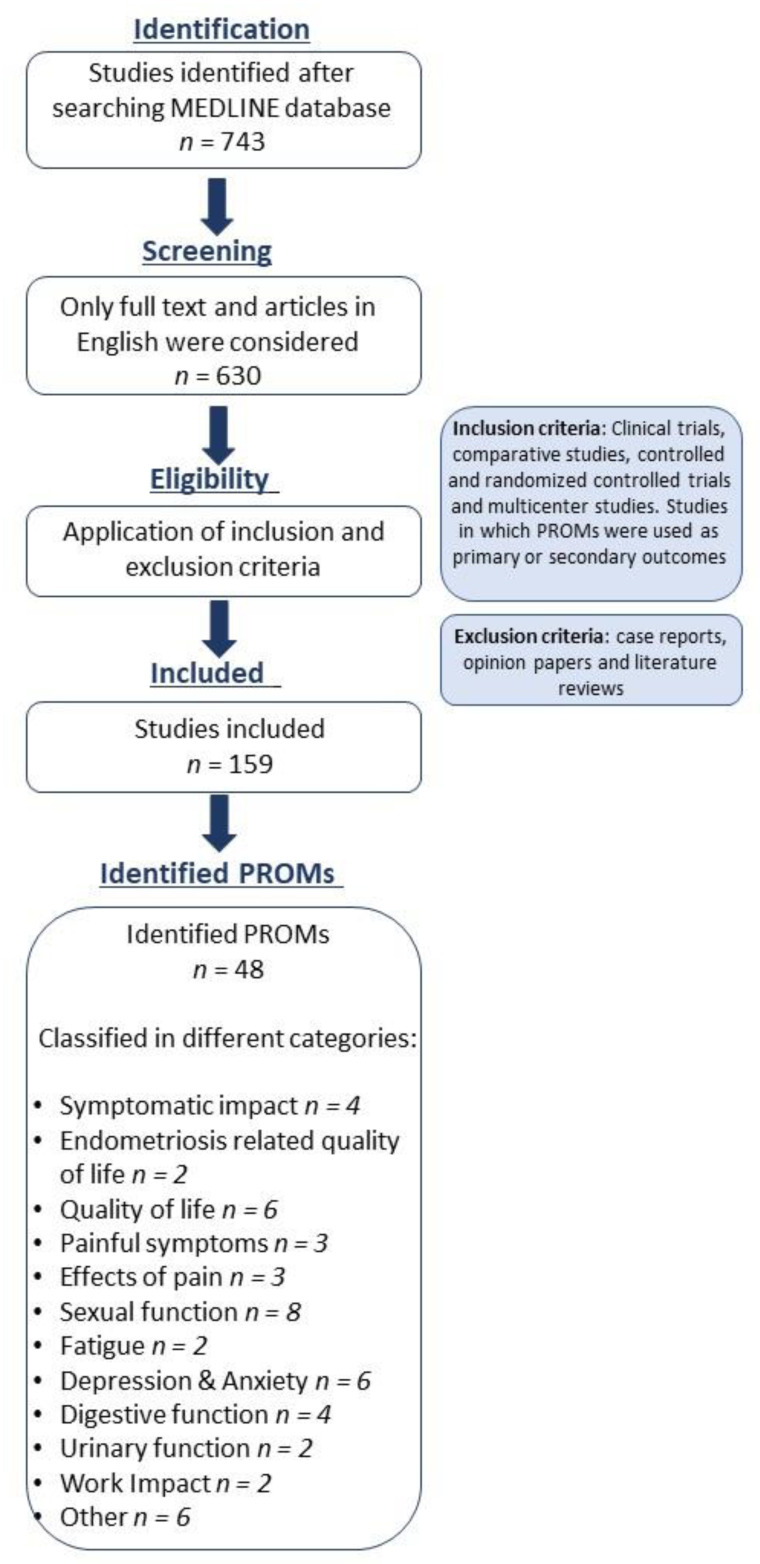
Flow diagram of the literature research.

We conducted a systematic literature review to identify the PROM tools employed in observational studies and clinical trials on endometriosis (Figure 1). A search of the MEDLINE/PubMed database was conducted for publications in English from January 1984 to January 2021. The search strategy was based on two search equations (algorithms), one dedicated to studies indexed with MeSH terms (Medical Subject Headings) and the other dedicated to studies not indexed at the time of the search, using exclusively free text. The MeSH terms used were: “quality outcomes”, “quality indicators”, “quality of life”, “patient reported outcomes”, and “endometriosis”. 

We initially identified 630 articles. The inclusion criteria for the analysis were (1) clinical trials, comparative studies, controlled and randomized controlled trials, and multicenter studies and (2) the use of PROMs in endometriosis as primary or secondary outcomes. The exclusion criteria were case reports, opinions, and literature reviews. After excluding duplicates and applying the inclusion/exclusion criteria, we identified 159 articles, 28 of which were clinical trials. All articles were reviewed to identify the PROMs employed in each study. 

The selected PROMs were then classified according to the type of outcome measured (general quality of life, endometriosis-related quality of life, symptomatic impact, painful symptoms, psychological effects of pain, sexual function, fatigue, depression and anxiety, digestive function, urinary function, work impact, and other), evaluated according to their reliability, validity, responsiveness to change, construct validity in a population of patients diagnosed with endometriosis, and unidimensionality. 

## 3. Results

We identified 48 different PROMs (Table 1), which provided measures for various outcomes. These PROMs were classified into separate categories depending on the type of outcomes they measured: (1) general quality of life, (2) endometriosis-related quality of life, (3) symptomatic impact, (4) painful symptoms, (5) psychological effects of pain, (6) sexual function, (7) fatigue, (8) depression and anxiety, (9) digestive function, (10) urinary function, (11) work impact, and (12) others.

### 3.1. PROMs Measuring General Quality of Life 

Numerous PROM tools that measure the general quality of life were identified and are summarized in Table 1. Most of these tools have not yet been validated in a population of patients diagnosed with endometriosis. 

The Short Form (SF-36) Health Survey is a measure of health status and was developed through the Medical Outcome Study (MOS) conducted by the RAND Corporation [16]. This PROM tool consists of 36 questions covering 8 health domains: (1) physical functioning, (2) physical role, (3) painful symptoms, (4) general health perceptions, (5) physical role functioning, (6) emotional role functioning, (7) social role functioning, and (8) mental health. Each domain is measured in a different subscale, and hence this PROM is multidimensional. The SF-36 is one of the main PROM tools for measuring quality of life and has been employed in numerous health economics studies. The current literature review identified the use of SF-36 in 53 endometriosis studies and clinical trials. It has also been adapted to its use in digital tools and its validity as an ePROM in an overall population has been proved [17]. The responsiveness, validity, and reliability of SF-36 has been validated for endometriosis [18]. This questionnaire is, however, complex to use due to the number of questions (36) and the heterogeneity of the questions labelling. The SF-12, a shorter version of the SF-36 (12 rather than 36 items) [19], covers mainly physical and mental health domains [20]. SF-12 has recently started to be employed in endometriosis-related studies due to being shorter and easier to use; however, its validity and reliability have not yet been validated in an endometriosis population. We identified 14 studies on endometriosis that used SF-12 as a PROM to measure quality of life.

The EuroQoL-5D (EQ-5D) is a questionnaire created by the EuroQoL group that has been used for the last 30 years in numerous clinical trials and research studies [21]. The EQ-5D is a PROM tool for assessing individual health-related quality of life (HRQoL) and also calculating quality adjusted life years (QALY), an indicator used in numerous health cost-utility analyses. The EQ-5D measures the generic health status and has two components: (1) health state description and (2) evaluation. In the description part, health status is measured in five dimensions: mobility, self-care, daily activities, pain/discomfort, and anxiety/depression. In the evaluation part, patients evaluate their overall health status using a visual analogue scale (EQ-VAS). Despite its generic nature, the tool’s validity and responsiveness has been validated in endometriosis [22]. A digital version of EQ-5D has been developed, tested, and validated in a general population [23].

The Patient-Reported Outcome Measurement Information System (PROMIS) Global Health is a 10-item generic PROM tool that measures two dimensions: global physical health and global mental health scores but has not been widely used in endometriosis studies (only one identified study [24]). A digital version of PROMIS global health has been developed, tested, and validated in a general population [25]. 

The World Health Organization Quality of Life Brief Version (WHOQOL BREF) is a quality-of-life assessment tool developed by the WHO [26] measuring four dimensions: (1) physical health, (2) psychological health, (3) social relationships, and (4) environment. The tool has been validated for endometriosis [27] but has not been employed much in studies (five identified studies). A digital version of WHOQOL BREF has been developed and validated in a general population [28].

The Nottingham Health Profile (NHP) evaluates patient health and its impact on daily life [29]. The NHP is a fairly long questionnaire divided into two parts: (1) 38 items covering six dimensions including physical abilities, pain, sleep, social isolation, emotional reactions, and energy level and (2) seven statements on areas of life commonly affected by health: employment, social life, personal relationships, sex life, hobbies, interests, and vacations. The NHP has not been validated for its responsiveness, reliability, and validity in endometriosis. 

### 3.2. PROM Measuring Endometriosis-Related Quality of Life 

The only PROM tool measuring endometriosis-related quality of life is the Endometriosis Health Profile (EHP), which is available in two formats: the long-form core instrument (EHP-30) and the short-form core version (EHP-5). The tool’s reliability and validity has been extensively demonstrated in endometriosis. 

The EHP-30 is a 30-item instrument that generates five dimension-scores covering pain, control and powerlessness, social support, emotional well-being, and self-image. There are optional modules covering other areas of health that might be relevant to patients with endometriosis (work, relationship with children, sexual relationship, feelings regarding the medical profession, feelings about treatments, and feelings about infertility) [30]. This questionnaire has interest in research since it allows for exploring separately different dimensions of the burden of endometriosis. Nonetheless, its lengthiness limits its use in routine care as well as its multidimensionality, which makes it difficult to apply in clinical decision making. 

The EHP-5 is a selection of items from the EHP-30 obtained by the dimensional reduction of the questions and includes one item from each of the five scales of the in EHP-30 [31]. The EHP questionnaires have been employed in numerous studies and clinical trials (e.g., NCT03981991 NCT02762461) [13,17]; in total, 57 studies using EHP tools were identified in the literature review. The EHP-5 has been fully validated in French in endometriosis patients [22], and it offers an unidimensional measurement of the endometriosis specific Qol impaction and has important responsiveness for endometriosis surgical or medical therapy. Both EHP-30 and EHP-5 are available in digital format. 

### 3.3. PROM Measuring Symptomatic Impact

We identified the PROMs used in the literature for measuring the symptomatic impact of endometriosis (Table 1). Out of the 43 identified PROMs, seven were specifically developed for endometriosis.

The Endometriosis Symptom Daily (ESD) and the Endometriosis Impact Scale (EIS) have recently been proposed as measures for measuring endometriosis-related symptoms on a daily and weekly basis [32]. These tools were developed following the FDA’s PROM guidelines [15] and its validation was carried out by a digital tool. The ESD is a patient-reported daily diary that assesses the key symptoms of endometriosis including pelvic pain severity, dysmenorrhea, vaginal bleeding, dyspareunia, and analgesia use. The EIS is a weekly electronic diary comprising items assessing the impact of endometriosis symptoms on patients’ daily lives over the past 7 days using a 5-point scale. The EIS measures the impact of endometriosis on patients’ physical activities, emotional well-being, and sexual activities. The ESD and EIS have been employed in non-interventional (NCT01643122) and interventional clinical studies (NCT02203331; NCT01822080). 

The Endometriosis Impact Questionnaire (EIQ) is a self-reported questionnaire that asks women about the impact of endometriosis on their lives over the “last 12 months”, “1 to 5 years ago”, and “more than 5 years ago”. The various measured items are ranked using a 5-point Likert scale. The various dimensions measure the physical, psychological, and social impact of the disease, as well as its impact on sexual and intimate relationships, fertility, employment, finance, education, and lifestyle. The EIQ was developed specifically for endometriosis, and its reliability (Cronbach alpha 0.98) and stability have been tested and validated [33]; however, its responsiveness to change has not been tested. To date, there have been no published studies that have used this PROM tool. 

The Menstrual Distress Questionnaire (MSQ) is a tool that measures cyclical perimenstrual symptoms, including the severity of menstruation-related lower abdominal pain, backpain, headache, and nausea/vomiting. The MSQ is a 46-item self-reported inventory for use in assessing and treating premenstrual and menstrual symptoms [34] and can distinguish cyclical from non-cyclical changes in physical symptoms, mood, behavior, and arousal. The MSQ is a generic women-health PROM tool and not endometriosis-specific. Two observational studies have been published using this tool [35,36]; however, none have validated this questionnaire for endometriosis.

### 3.4. PROMs Measuring Painful Symptoms 

Pelvic pain is one of the main symptoms of endometriosis. There are several types of pelvic pain: severe dysmenorrhea, deep provoked dyspareunia, and non-menstrual chronic pelvic pain. Other symptoms may include cyclical painful defecation or bladder pain. To evaluate the clinical improvement of patients with endometriosis, it is essential to measure the pain. Measurement may include self-perceived intensity, location, and description of the patient [37]. Various PROM tools have been identified in endometriosis-related studies (Table 1). 

The Endometriosis-Associated Pelvic Pain (EAPP) is a unique visual analogue scale (VAS) that measures pain with a scale ranging from no pain to extreme pain, the worst imaginable [38]. It refers to “your endometriosis pain” that may relate to several distinct contents (unfortunately not the same across the women). Despite this lack of specificity, the EAPP is one of the main tools used in clinics and is considered the “gold standard” for measuring pain in endometriosis due to its simplicity. We identified 23 studies that employed this tool to measure pain in endometriosis. 

To respond to this criticism, our group designed, according the FDA process of PROMs specifically developed for measuring the entire pain experience in endometriosis, ENDOPAIN-4D, which measures the painful symptoms of endometriosis in a comprehensive and multidimensional manner and includes the patients’ perspective. ENDOPAIN-4D considers four dimensions: (1) spontaneous pelvic pain and dysmenorrhea, (2) dyspareunia, (3) painful bowel symptoms, and (4) other symptoms. This questionnaire has the ability of describing the pain by its distinct dimensional aspects using the calculation of four above sub scores. It may also evaluate the pain as a whole by calculating an aggregated score which was hugely correlated to the EAPP [39]. The questionnaire evaluates the complex nature of the experience of pain in endometriosis and is the only PROM tool for evaluating pain that has been developed specifically based on the testimony of patients with endometriosis [40,41].

The PainDETECT questionnaire (PD-Q) is a simple and reliable screening questionnaire for neuropathic pain [42] and consists of seven questions that address the quality of neuropathic pain symptoms. The PD-Q has been used in clinical trials as an indicator of improved quality of life in endometriosis (NCT04081532), despite the fact that it has not been validated for endometriosis. It may be useful in routine use to search for a neuropathic component of the endometriosis-related pain. Neuropathic pain is not rare in native endometriosis patients or after surgery [43].

### 3.5. PROMs Measuring Psychological Effects of Pain 

The Pain Catastrophizing Scale (PCS) quantifies a patient’s pain experience through questions on how they feel and what they think about when they are in pain [44]. Unlike other tools, the patient does not need to be feeling pain while completing the scale. The PCS has been used in various studies in endometriosis; however, its validity and reliability have not yet been demonstrated in this context. This measurement might have an interest in routine care to improve the pain by addressing the patient for specialized pain management. 

The Pain Vigilance and Awareness Questionnaire (PVAQ) measures the degree of pain vigilance [45] and is a 16-item list that asks patients about their focus on pain during the last 2 weeks. Despite not being validated for endometriosis, the PVAQ has demonstrated good psychometric properties in measuring pain in patients with chronic pain syndromes such as fibromyalgia [46].

The Pain Anxiety Symptom Scale (PASS) measures anxiety caused by pain [47]. The measured indicators are fear of pain, cognitive anxiety, avoidance behavior, and physiological anxiety symptoms, which are measured on a 6-point scale. Few studies have used this tool in the context of endometriosis; however, there has been no published psychometric validation showing that the PASS is an appropriate tool for use in endometriosis.

### 3.6. PROMs Measuring Sexual Function

The sexual function of women with endometriosis can be altered not only by the disease-induced pain, but also by the various treatments undergone. Considering sexual function as an important outcome in endometriosis is essential. In 2016, Fritzer et al. indicated that the sexual quality of life in endometriosis was rarely considered and measured with standard tools [48].

The PROMs employed in the literature to measure sexual function are summarized in Table 1; however, none of these tools were specifically developed for evaluating sexual function in patients with endometriosis. 

The Female Sexual Function Index (FSFI) is a multidimensional tool that measures the sexual function of women [49] in six dimensions including desire, arousal, lubrication, orgasm, satisfaction, and pain. The FSFI is the most widely used PROM questionnaire for measuring sexual function in endometriosis studies and is often used together with the Female Sexual Distress Scale (FSDS), which evaluated personal distress due to problems related to sexual function [50].

The Sexual Activity Questionnaire (SAQ) is a PROM initially developed to study the impact of tamoxifen on the sexual functioning of women [51]. Its use in the context of endometriosis has, however, been psychometrically validated [52]. The SAQ assesses various aspects of sexual function including pleasure, desire, satisfaction, vaginal dryness, penetration pain, and intercourse frequency. Its unidimensionality has been proved in a population of patients diagnosed with endometriosis [52]. 

The Sexual Health Outcomes in Women Questionnaire (SHOW-Q) is often used to evaluate sexual function [53] and was initially designed to evaluate the impact of pelvic problems on sexual desire, intercourse frequency, satisfaction, orgasms, and discomfort. Most of the identified PROM tools have been designed to measure sexual activity in cases of having a male partner only. It is important to underline that SHOW-Q’s inclusive aspect was also designed to eventually measure sexual activity in women without a male partner (patients without a usual partner or homosexuals). 

The Golombok Rust Inventory of Sexual Satisfaction (GRISS) measures sexual function and sexual satisfaction [54] and has been designed for women and men, which helps to compare the impact of the disease on both members of the couple. It is interesting to use in clinical practice because it offers a one-dimensions score. However, certain questions are obsolete and inappropriate for certain gynecologic conditions such as endometriosis, which explains why it has not been used much in studies. 

The Short Sexual Functioning Scale (SSFS) is a questionnaire consisting of four items that address points in sexual dysfunction: decreased/increased sexual desire, dry vagina, orgasmic dysfunction, and pain during intercourse. It was developed specifically to use in a population of patients diagnosed with endometriosis and has proved to have good reliability and internal consistency [55].

Derogatis Sexual Functioning Inventory (DSFI) is a multidimensional PROM tool that measures various aspects of psychological and sexual function. It has 10 different subscales: information, experiences, drive, attitudes, psychological symptoms, affects, gender role definition, fantasy, body image, and sexual satisfaction [55]. Two different index can be calculated using this inventory: the sexual functioning index (SFI) and the Global sexual satisfaction index (GSSI). Both subscales have been used independently since they provide unidimensional scores in endometriosis studies [56].

The Sexual Self-Conscious Scale (SSCS) is a 12-item PROM tool, validated as a self-report instrument for assessing the private and public aspects of proneness in sexual situations and of sexual anxiety and discomfort. It has two subscales: embarrassment and self-focus [57].

### 3.7. PROMs Measuring Fatigue

Endometriosis alters other aspects of daily life including fatigue. Studies have shown that patients with a symptomatic course of endometriosis present higher stress levels and fatigue in their daily life [58]. Only two PROM tools were identified in the search: the PROMIS Fatigue Short Form and the Piper Fatigue Scale (Table 1).

The PROMS Fatigue Short Form was initially created to measure fatigue in the general healthy population [59]; however, it has been psychometrically validated in an endometriosis population [60]. The Short Form measures fatigue and fatigue-related issues with a recall period of the last 7 days.

The Piper Fatigue Scale measure four dimensions related of subjective fatigue: behavioral/severity, affective meaning, sensory, and cognitive/mood [61]. The scale has been validated for patients with breast cancer [62]; however, it has not been validated in endometriosis.

### 3.8. PROMs Measuring Depression and Anxiety

The impact of endometriosis on women’s mental health is a critical aspect of the disease management and is often under considered. Studies have shown that women with endometriosis often suffer from depressive and anxiety disorders [63]. This literature review identified several PROMs that measure depression and anxiety (Table 1).

The Hospital Anxiety and Depression Scale (HADS) is a generic questionnaire used for numerous conditions to evaluate anxiety and depression [64]. Its use in daily practice is starting to be seen for other conditions (e.g., cancer) [65] since it can be used as both a one-dimensional measure (of major depression) or as separate depression and anxiety indexes. HADS has often been employed in endometriosis despite the fact that it has not been psychometrically validated. Different studies have shown the adaption of this PROM to its electronic version [66].

The Beck Depression Inventory (BDI) is a 21-item self-reported questionnaire that assesses the severity of depression in adults (one-dimension scale). The BDI has demonstrated excellent reliability and internal consistency in a primary care population; however, its psychometric properties have not been validated in an endometriosis population despite the fact that it is often used in clinical research on endometriosis [67].

The Patient Health Questionnaire (PHQ-9) is a unidimensional depression scale for diagnosing depressive and other mental disorders [68]. The PHQ-9 has been validated in an endometriosis population, proving its good psychometric properties [69]. Its use in routine care using a digital tool has been proved in neurologic patients [70].

The Spielberger State-Trait Anxiety Inventory (STAI) is an instrument that measures anxiety from the perspective of states versus traits (two dimensions) [71]. The state measurement assesses an individual’s feelings at the time of the evaluation. The trait anxiety measurement focuses on the individual’s general feelings. The practical benefits of distinguishing between traits and states have long been debated. Nevertheless, this PROM has been used in the context of endometriosis, although its validity in this disease has yet to be proven.

The General Anxiety Disorder-7 (GAD-7) scale is an instrument with an unidimensional structure that measures the severity of generalized anxiety disorders (pain disorders, social anxiety disorder, and posttraumatic stress disorder) [72]. The GAD-7 has been validated in an endometriosis population, proving its good psychometric properties [69].

The Beck Anxiety Inventory (BAI) measures common somatic and cognitive symptoms of anxiety in adults and adolescents [73].

### 3.9. PROMs Measuring Digestive Function

Deep infiltrating endometriosis is often associated with digestive malfunction including painful symptoms or alteration of digestive function as bowel movement [74]. These symptoms are important in clinical decision making among women with intestinal endometriosis. Numerous studies, especially those exploring the outcomes of surgery, have measured digestive outcomes.

The Gastrointestinal Quality Life Index (GIQLI) is a self-administered questionnaire including 36 questions measuring five dimensions of digestive function: digestive symptoms, physical status, emotions, social dysfunction, and the effects of medical treatment [75]. It does not only include questions on gastrointestinal symptoms, but also other aspects related to the quality of life (physical well-being, mental well-being, digestion, and defecation) validated in various gastrointestinal diseases.

The Knowles-Eccersley-Scott-Symptom (KESS) scoring system evaluates constipation [76], measuring 11 items: duration of constipation, laxative use, bowel movement frequency, unsuccessful evacuation attempts, feeling of incomplete evacuation, abdominal pain, bloating, enemas, time taken, difficulty evacuating, and stool consistency.

The Fecal Incontinence Quality of Life (FIQL) is a disease-specific tool composed of a total of 29 items that evaluate the impact of fecal incontinence on four dimensions of patients’ quality of life: lifestyle, coping behavior, depression or self-perception, and embarrassment [77].

The Wexner scoring system assesses anal incontinence by cross-tabulating frequencies and various anal incontinence presentations [78].

The GIQLI and the KESS are the most widely used PROMs for evaluating digestive function in clinical studies.

### 3.10. PROMs Measuring Urinary Function

The International Consultation on Incontinence Questionnaire-Female Lower Urinary Tract Symptoms (ICIQ-FLUTS) evaluates female lower urinary tract symptoms and their impact on quality of life by measuring three distinct dimensions: filling symptoms, voiding symptoms, and incontinence symptoms [79].

### 3.11. PROMs Measuring Work Impact

The Health-Related Productivity Questionnaire (HRPQ) measures the impact of the disease on work productivity and daily chores [80]. This generic questionnaire has been psychometrically validated for endometriosis [81].

The Work Productivity and Activity Impairment Questionnaire (WPAI-SHP) measures the effect of general health and symptom severity on work productivity and regular activities over 7 days [82]. The WPAI-SHP covers absenteeism, presenteeism, overall work impairment, and activity impairment, and it allows measuring eight different scores. The WPAI-SHP has already been used in a digital tool developed by NHS Wales that allows the collection of electronic PROMs at home in a general population [83].

### 3.12. Other PROMs Identified in Endometriosis

The International Fitness Scale (IFS) measures physical fitness, cardiorespiratory fitness, muscular fitness, speed-agility, flexibility, and overall fitness [84].

The Coping Strategies Inventory (CSI) assesses the ability to cope with stress and difficulties [85]. The CSI has 72 items covering eight main dimensions: problem solving, cognitive restructuring, social support, express emotions, problem avoidance, wishful thinking, social withdrawal, and self-criticism.

FertiQoL is a tool that measures the influence of fertility problems on quality of life and includes general health, self-perception, emotions, partnership, family, social relationships, work life, and future life plans [86]. It includes 36 items that assess core (24 items) and treatment-related quality of life (10 items) and overall life and physical health (2 items). It measures nine different dimensions. FertiQoL has been specifically designed for an endometriosis population [86].

Quality of sleep can also be affected in patients with endometriosis. Sleep dysfunction is prevalent in patients who have chronic pain conditions [87] and has been strongly associated with dysmenorrhea [88] and chronic pelvic pain [89]. We identified three separate PROMs that measure sleep quality.

The Pittsburg Sleep Quality Index (PSQI) evaluates sleep quality and disturbance over a 1-month interval [90]. The PSQI measures the following dimensions: subjective sleep quality, sleep latency, sleep duration, habitual sleep efficiency, sleep disturbances, use of sleeping medication, and daytime dysfunction.

The Insomnia Severity Index (ISI) screens for insomnia [91] and focuses on the subjective qualities of patients’ sleep patterns, the degree to which insomnia affects daily functioning, how noticeable the insomnia is to others, and the overall level of distress created by the insomnia.

The Epworth Sleepiness Scale (ESS) evaluates the chances of dozing off or falling asleep while engaged in various activities [92]. Its unidimensionality has been confirmed in a large diverse clinical population [93].

**Table 1:** List and classification of patient-reported outcome measures (PROMs) used in endometriosis.

PROM Tool	Reference	Indicators Measured	Disease Specificity	Responsiveness	Reliability (Cronbach Alpha)	Construct Validity	Dimensions	Digitalization	Ref Of Studies Used
*Symptomatic Impact*									
Endometriosis Symptom Diary (ESD)	[32]	Pelvic pain severity, dysmenorrhea, vaginal bleeding, dyspareunia, analgesic use	Yes	-	-	-	5	Yes	NCT01822080
Endometriosis Impact Scale (EIS)	[32]	Physical activities, emotional well-being, and sexual activities	Yes	-	-	-	10	Yes	NCT0220331
Endometriosis Impact Questionnaire (EIQ)	[33]	Physical-psychosocial, fertility, sexual employment, educational, and lifestyle	Yes	Yes	0.99	Yes	10	No	[33]
Menstrual Distress Questionnaires (MDQ)	[34]	Lower abdominal pain, back pain, headache, and nausea/vomiting	-	-	-	-	4	No	[35]
*Endometriosis-specific Quality of Life*									
Endometriosis Health Profile (EHP-5)	[31]	Pain, control and powerlessness, emotional well-being, social support, self-image	Yes	Yes	0.83	Yes	1	Yes	[94,95,96,97]
Endometriosis Health Profile (EHP-30)	[30]	Pain, control and powerlessness, emotional well-being, social support, self-image	Yes	Yes	0.83	Yes	6	Yes	[35,98,99,100,101,102,103]
*Quality of life*									
Short Form Health Survey (SF-12)	[19]	Physical functioning, social functioning, limitations due to physical problems, limitations due to emotional problems, mental health, energy and vitality, pain, and general perception of health	No	-	-	-	2	No	[100,104,105,106]
Short Form Health Survey (SF-36)	[16]	Physical functioning, physical role, bodily pain, general health, vitality, social functioning, emotional role, mental health	No	Yes	0.75	Yes	8	No	[103,107,108,109,110,111]
EQ-5D	[21]	Pelvic pain severity, dysmenorrhea, vaginal bleeding, dyspareunia, analgesic use	No	Yes	-	Yes	5	Yes	[112,113]
PROMIS Global Health	[59]	Global physical health, global mental health	No	-	-	-	2	Yes	[60]
World Health Organization Quality of Life (WHOQoL BREF)	[26]	Physical health, psychological health, social relationships, environment	No	Yes	0.83	Yes	4	Yes	[27,114,115,116]
Nottingham Health Profile (NHP)	[29]	Physical abilities, pain, sleep, social isolation, emotional reactions, energy level, and disability	No	-	-	-	6	No	[117]
*Painful symptoms*									
Endometriosis Associated Pelvic Pain (EAPP)	[38]	Pain intensity	Yes	-	-	-	1	No	[38,118,119]
PainDETECT questionnaire (PD-Q)	[42]	Pain	No	-	-	-	1	No	[118]
ENDOPAIN-4D	[40]	Pain symptoms	Yes	Yes	0.61	Yes	1	No	[41]
*Effects of pain*									
Pain Catastrophizing Scale (PCS)	[44]	Pain impact	No	-	-	-	1	No	[102,103]
Pain Vigilance and Awareness Questionnaire (PVAQ)	[45]	Pain	No	-	-	-	1	No	[103]
Pain Anxiety Symptom Scale (PASS)	[47]	Pain	No	-	-	-	1	No	[103]
*Sexual Function*									
Female Sexual Function Index (FSFI)	[49]	Sexual Function	No	-	-	-	6	No	[98,101,102,108]
Sexual Activity Questionnaire (SAQ)	[51]	Sexual Function	No	Yes	0.78	Yes	1	No	[52]
Sexual Health Outcomes in Women Questionnaire (SHOW-Q)	[53]	Sexual Activity—satisfaction, orgasm, desire, and pelvic problem interference	No	-	-	-	1	No	[119]
Golombok Rust Inventory Sexual Satisfaction (GRISS)	[54]	Sexual Function	No	-	-	-	1	No	[116]
Female Sexual Distress Scale (FSDS)	[50]	Sexual Distress	No	-	-	-	6	No	[108]
Short Sexual Functioning Scale (SSFS)	[55]	Sexual Disfunction	Yes	Yes	0.84	Yes	1	No	[55]
Derogatis Sexual Functioning Inventory (DSFI)	[120]	Psychological and sexual function	No	-	-	-	10	No	[56]
Sexual Self-Conscious Scale (SSCS)	[57]	Proneness to sexual situations	No	-	-	-	2	No	[57]
*Fatigue*									
PROMIS Fatigue Short Form	[59]	Fatigue	No	Yes	0.93	Yes	1	Yes	[121]
Piper Fatigue Scale	[61]	Fatigue	No	-	-	-	4	No	[102]
*Depression & Anxiety*									
Hospital Anxiety and Depression Scale (HADS)	[64]	Anxiety and Depression	No	-	-	-	1	Yes	[102,105,122]
Beck Depression Inventory (BDI)	[123]	Depression	No	-	-	-	1	No	[97,124]
PHQ-9	[68]	Depression	No	-	-	-	1	Yes	[122]
Spielberger State-Trait Anxiety Inventory (STAI)	[71]	Anxiety	No	-	-	-	2	No	[110]
General Anxiety Disorder scale (GAD-7)	[72]	Anxiety	No	-	-	-	1	No	[125]
Beck Anxiety Inventory	[73]	Anxiety	No	-	-	-	1	No	[97,124]
*Digestive function*									
Gastrointestinal Quality Life Index (GIQLI)	[75]	Digestive symptoms, physical status, emotions, social dysfunction, and effects of medical treatment	No	-	-	-	5	No	[74,102,126]
Knowles-Eccersley-Scott-Symptom (KESS)	[76]	Constipation	No	-	-	-	1	No	[127,128]
Fecal Incontinence Quality of Life	[77]	Fecal Incontinence	No	-	-	-	5	No	[126]
Wexner	[78]	Fecal Incontinence	No	-	-	-	1	Yes	[126]
*Urinary function*									
Urinary Symptom Profile (USP)	[129]	Urinary Function	No	-	-	-	1	No	[128]
International Consultation on Incontinence Questionnaire-Female Lower Urinary Tract Symptoms (ICIQ-FLUTS)	[130]	Fatigue	No	-	-	-	3	No	[131]
*Work impact*									
Health-Related Productivity Questionnaire (HRPQ)	[80]	Impact of disease on absenteeism and presenteeism for employment and household chores	No	Yes	-	Yes	1	No	[121]
Work Productivity and Activity Imparity (WPAI)	[82]	Absenteeism, presenteeism (overall work impairment and activity impairment).	No	-	-	-	6	No	[100]
*Others*									
International Fitness Scale	[84]	Perceived physical fitness	No	-	-	-	6	No	[132]
Coping Strategies Inventory	[133]	Problem solving, cognitive restructuring, social support, express emotions, problem avoidance, wishful thinking, social withdrawal, and self-criticism	No	-	-	-	14	No	[97]
FertiQoL	[86]	Infertility-related quality of life	No	-	-	-	9	No	[134]
Pittsburg Sleep Quality Index	[90]	Sleep quality	No	-	-	-	7	No	[102,135,136]
Insomnia Severity Index	[91]	Sleep quality	No	-	-	-	1	No	[135]
Epworth Sleepiness Scale	[92]	Sleep quality	No	-	-	-	1	No	[135]

## 4. Discussion

In this literature review, we identified 48 PROM tools that have been used to evaluate various dimensions of the impact of endometriosis on patients. PROM tools have already been extensively used in clinical studies and their use has been shown to alert patients of issues to discuss with their clinicians, to help both patients and clinicians track health outcomes over time, and to facilitate comparisons of different patient groups with the same conditions. The routine use of PROMs in the context of endometriosis care would facilitate patient-provider communication and the implementation of patient-centered care, in addition to providing a tool for patients´ self-management of the disease.

### 4.1. PROMs in Routine Clinical Care

The identification of these 48 PROMs is a first step to creating a more limited standard set of PROM questionnaires to be used in routine endometriosis care. Using PROMs in routine clinical care can contribute to a shift towards a more patient-centered care [137]. The use of PROMs in endometriosis routine clinical care has the potential to improve clinician-patient communication [138] by: (1) helping patients raise certain concerns with their clinicians, (2) helping clinicians sensitize patients to health issues related to their underlying health condition and treatment, (3) enable comparisons to be made between an individual patient’s outcome with those of other patients with equivalent health conditions, and (4) promote shared-decision making when possible, based on information on effects of alternative treatments on PROMs [136]. To facilitate PROM use in clinical practice, PROM data must be made available to clinicians and patients in a simple format. To do so, the use of digital tools is essential for its successful implementation. The validation of PROM tools in digital format is important in order to properly use them. Simplification of the use of PROM tools can be achieved using patient-centered dashboards that can be designed in order to be used by clinicians and patients during consultation and to support decision making. To be successful, we must consider a variety of issues from both the clinician and patient perspectives. These issues include how and when the PROM data are collected, from whom it is collected (e.g., patient groups by age or ethnicity), and the most acceptable and efficient collection method (paper, electronic, telephone, etc.) for those groups.

The selected tools should also capture the patient-reported information relatively quickly and feasibly, so as not to overburden the respondents and to ensure that clinicians receive the information in a timely manner [139]. The patients must be willing and able to provide the information. Endometriosis symptomatology is multifactorial and it changes over time based on a number of factors including patient life experiences, perceptions, and medical treatment. These changes require agility on the part of clinicians and patients to respond appropriately, and documentation over time to detect patient-specific patterns, both of which are primary goals of the envisioned patient-centered dashboards for endometriosis.

### 4.2. Disease-Specific vs. Generic PROMs

A weakness in the group of 48 PROMs we identified is that only 13 have been tested and their content validated for use specifically with endometriosis: the ESD, EIS, EIQ, EHP-5, EHP-30, EQ-5D, SAQ, SFSFS, WHOQoL, HRPQ, PROMIS Fatigue, EAPP, and the ENDOPAIN 4D. These PROMs have either been created starting from testimonies from patients suffering with endometriosis, making it condition specific by default or generic PROMs that have been validated in a population diagnosed with endometriosis. The validation of PROM instrument is based on demonstrating the disease-specificity, reliability, responsiveness, and sensitivity to change from a patient’s perspective [140].

However, combinations of generic and condition-specific PROMs have been employed in the context of endometriosis and in other treatment areas as well. Kyte et al. reported on the use of PROMs in physiotherapy, specifically for patients with severe arm paresis after stroke [141]. They used a combination of a generic PROM to measure the patients’ independence in activities of daily living, with a specific PROM that measures quality of life issues in stroke patients. These measurements were added to an additional outcome measure that evaluated the patients’ recovery from a functional perspective. The combined use of these measures helped ensure that the patients’ perspectives are appropriately captured in addition to traditional clinical outcome measures, and a similar multi-pronged approach seems most appropriate for patients with endometriosis.

In terms of conditions closer to endometriosis, Moss et al. performed a literature review of 21 studies specifically on the use of PROMs in pelvic abdominal cavity, with the aim of determining both oncological benefits and the impact of the use of PROMs on patients’ experiences [142]. The authors found that the use of PROMs not only led to improved outcomes, but also enhanced patient-provider communication as well as patient satisfaction with the care process.

In the UK, a qualitative study is underway by Anderson, et al., in which the use of PROMs for patients with end-stage kidney disease treated with hemodialysis (HD) is being explored in a similar manner as envisioned for endometriosis care [101]. Patients with end-stage kidney disease treated with HD also have complexity in symptomatology, patient and clinician perceptions, and quality of life, as do patients with endometriosis. The authors’ intention is to use the results of their research to inform future studies on PROMs in the clinical setting beyond HD. They also hope to contribute to the development of PROMS specifically aimed at enhancing quality of life for patients as well as to improve service delivery and patient care.

Therefore, the fact that only 13 of the PROMs we identified have been designed specifically for endometriosis does not mean the remaining PROMS are not useful for our patient-centered dashboards or for the ultimate care improvement goals envisioned.

### 4.3. PROM Validity and other Essential Aspects

In addition to the importance of ease of use mentioned earlier, PROM aspects such as content validity and construct validity, responsiveness, generalizability, and feasibility are essential to consider. Validity, specifically construct validity, refers to the ability of the tool to measure what it intends to measure from a patient perspective In addition to the seven tools specifically designed for endometriosis, 7 of the 43 PROMs identified in this review were determined to be responsive and to have construct validity despite not being disease-specific: the SF-12, EQ-5D, the WHOQoL BREF, SAQ, SFSS, PROMIS Fatigue short form, and the HRPQ. These tools might be particularly useful in conjunction with those PROMs that have been validated for endometriosis.

Content validity is established based on the relationship between the content or items in the tool and the concept it is supposed to measure [143]. It represents the intersection between the characteristics of the disease and the patient’s life experience. An appropriately designed tool in terms of content validity will be responsive or sensitive to change when assessing treatment differences between patient groups.

Furthermore, the relationship between symptoms and quality of life impairment is complex. All of these parameters are important in endometriosis care and it is tempting to speculate that they might be directly related. However, what is demonstrated clinically is often different from what is perceived as problematic by patients in their everyday lives [10]; thus, symptom severity alone does not adequately assess the effects of endometriosis on an individual’s life. To adequately assess these effects, symptom or generic/condition-specific PROM tools can be used along with clinical measures such as imaging and lab results.

Thus, determining a useful subset of the identified PROMs will offer measurement tools validated specifically for endometriosis along with a variety of relevant and feasible instruments that have been shown to assess the impact of the disease on patients from various personal perspectives.

The next step will be to assess the most valuable of the 43 PROMs via qualitative and quantitative study among patients and clinician followed by the development of the patient-centered dashboards. The dashboards will be part of a feasibility study to determine how the PROMS are used by patients and clinicians in the care and self-management processes, followed by a randomized study to assess how well the dashboards actually improve care, patient satisfaction, and outcomes. The selection of the specific indicators that will be included in the dashboard will be the object of a future study; nevertheless, considering the analysis and the literature review we performed, we can confirm that this future dashboard will include measurements regarding pelvic pain, endometriosis specific quality of life, impact in work and absenteeism, sexuality, and for specific patients the impact in the digestive function.

## 5. Conclusions

Defining and integrating a standard set of PROMs in routine endometriosis care will serve to help evaluate the impact, appropriateness, quality, and performance of health care in this disease context. In addition to providing a decision support tool to patients and clinicians via the dashboards, we will be able to facilitate the comparison of results across treatments and care pathways, sharing best practices between professionals and leading to continuous improvements in endometriosis care.
